# Four-quadrant analysis of universal health coverage progress in the Western Pacific region: facilitators, barriers, and trajectories to 2030

**DOI:** 10.1080/16549716.2026.2659459

**Published:** 2026-04-21

**Authors:** Yiran Tian, Jin Xu, Mingyue Li, Haoqing Tang, Huixian Zheng, Ren Long, Leyang Han, Daihan Li, Yiting Lin, Yaoyun Zhang, Chengkun Lv, Xiaoyun Liu

**Affiliations:** a China Center for Health Development Studies, Peking University, Beijing, China; b Beijing Institute for Health Development, Peking University, Beijing, China; c National Health Commission Key Laboratory of Health System Reform and Governance, Peking University, Beijing, China; d WHO Collaborating Center for Universal Health Coverage, Peking University, Beijing, China

**Keywords:** Service coverage, financial hardship, universal health coverage, health system strengthening, Western Pacific Region

## Abstract

**Background:**

Universal health coverage (UHC) progress in the Western Pacific Region (WPR) varies across countries and between service coverage (SDG 3.8.1) and financial hardship (SDG 3.8.2).

**Objectives:**

To characterize patterns and trajectories of UHC progress in the WPR, identify associated health-system and socioeconomic factors, and project progress to 2030.

**Methods:**

This convergent mixed-methods study used data from the WHO, World Bank, and UN. Countries were classified according to a four-quadrant typology, with 2022 global estimates of SDG 3.8.1 and 3.8.2 serving as reference benchmarks. Fixed-effects panel models were employed to examine associations of SDG 3.8.1 with key covariates. Peer-reviewed, policy, and grey literature were analyzed using the READ approach and thematic framework analysis, while Bayesian models were used to project UHC progress to 2030.

**Results:**

From 2000 to 2022, SDG 3.8.1 in the WPR rose from 63 to 81, while SDG 3.8.2 fell from 39.6% to 27.0%. Twelve countries were classified as low SDG 3.8.1/low SDG 3.8.2, five as high/low, three as low/high, and China alone as high/high. More favorable patterns were associated with stronger financing, primary health care orientation, workforce capacity, and governance, whereas less favorable patterns were linked to fragmented financing, workforce shortages, remoteness, climate vulnerability, and population ageing. SDG 3.8.1 was positively associated with education and urbanization and showed an inverted U-shaped relationship with GDP per capita. By 2030, SDG 3.8.1 and SDG 3.8.2 are projected to reach 85.6% and 22.2%, respectively.

**Conclusion:**

Accelerating progress will require context-specific reforms tailored to individual countries.

## Paper context

- **Main findings:** Universal health coverage in the Western Pacific Region improved overall between 2000 and 2022, but progress remained highly uneven across countries, with different combinations of service coverage and financial hardship shaped by distinct health system and socioeconomic determinants.
- **Added knowledge:** By integrating regional trends, country trajectories, quantitative analysis, documentary evidence, and projections, this study provides a more explanatory account of why universal health coverage progress has diverged across countries in the Western Pacific Region.
- **Global health impact for policy and action:** Accelerating progress towards universal health coverage will require context-specific policy responses that prioritize high-impact interventions, improve equitable and efficient resource allocation, and address whether the main constraint in each setting is limited service coverage, persistent financial hardship, or both.

## Background

‘Leaving no one behind’ is a central promise of the 2030 Agenda for Sustainable Development. Target 3.8 of the Sustainable Development Goals (SDGs) calls for the achievement of universal health coverage (UHC), ensuring that all people can access the quality health services they need without suffering financial hardship [1]. WHO and the World Bank’s UHC monitoring framework emphasizes simultaneous assessment of service coverage index (SCI, SDG 3.8.1) and financial hardship (FH, SDG 3.8.2). Joint assessment is essential because these two dimensions of UHC do not necessarily move in parallel, and gains in service coverage may not be matched by improvements in financial protection, while low financial hardship may also reflect unmet need or forgone care rather than true financial protection [2].

Globally, the world has made progress since 2000 on both dimensions of UHC, with SDG3.8.1 rising from 54 in 2000 to 71 in 2022, and SDG3.8.2 falling from 34% to 26%. Since 2015, progress towards UHC has been slower than in previous years, suggesting that the world is unlikely to achieve its 2030 UHC targets without accelerated gains [3]. As one of WHO’s six regions, the Western Pacific Region (WPR) includes 38 countries and areas and covers a population of nearly 2.2 billion people [4,5]. The region is highly diverse in population size, economic development, and health-system capacity [6].

Existing research has advanced UHC measurement and policy analysis, but important gaps remain. Much of the literature has focused on single-country analyses [7,8], on only one dimension of UHC [9], or on context-specific adaptations of monitoring frameworks [10,11], with less attention to how service coverage and financial protection evolve together across countries and over time, particularly in highly heterogeneous regions such as the WPR. To address this gap, this study applies a four-quadrant framework and integrates quantitative and qualitative evidence to examine uneven UHC progress across the WPR and its underlying drivers, and assess likely trajectories to 2030.

## Methods

### Data sources and study sample

Data were collected from WHO, World Bank, and United Nations databases (Appendix 1). SDG 3.8.1, the UHC service coverage index, is constructed from 14 tracer indicators grouped into four sub-indices: reproductive, maternal, newborn, and child health (RMNCH), infectious diseases, noncommunicable diseases (NCDs), and service capacity and access [12]. SDG 3.8.2 is defined as the proportion of the population incurring out-of-pocket health spending exceeding 40% of household capacity to pay, encompassing both impoverishing and substantial non-impoverishing out-of-pocket health spending [13].

## Four-quadrant classification and trajectory analysis

The four-quadrant framework was used as a descriptive comparative tool to examine patterns of UHC positioning across countries and areas in the WPR. Quadrant boundaries were defined using the official 2022 global estimates for SDG 3.8.1 and SDG 3.8.2, reported by WHO, so as to provide an externally defined and internationally comparable benchmark. Countries and areas were grouped into four categories relative to the 2022 global reference values: lower SDG 3.8.1 and lower SDG 3.8.2; higher SDG 3.8.1 and lower SDG 3.8.2; lower SDG 3.8.1 and higher SDG 3.8.2; and higher SDG 3.8.1 and higher SDG 3.8.2. Because SDG 3.8.2 measures financial hardship, lower values were interpreted as better and higher values as worse.

To compare the pace of change between the Millennium Development Goals (MDGs) period and the Sustainable Development Goal (SDGs) period, annualized rates of change (ARC) were calculated for selected indicators using the standard log-linear formulation commonly applied in WHO trend assessments [14]. For each country or regional series, ARC was estimated as:

$$\mathrm{ARC}\left(\%\right)=\frac{\ln\left(\frac{Y_{t_{2}}}{Y_{t_{1}}}\right)}{t_{2}-t_{1}}\times 100$$

where $Y_{t_{1}}$ and $Y_{t_{2}}$ denote the observed indicator values at the beginning and end of the specified period, and $t_{2}-t_{1}$ denotes the number of years between the two observations. ARC was calculated separately for 2000–2014 and 2015–2022 to compare changes between the MDGs and SDGs eras.

## Evidence reviews and thematic analysis

To interpret quantitative findings and differences across quadrant profiles, an explanatory evidence review integrated with structured document analysis was conducted [15,16]. This component was designed to support interpretation of the four-quadrant typology and was not intended as a formal systematic review or meta-analysis [17]. Peer-reviewed literature was identified through structured searches of PubMed, Web of Science, and Scopus, with Google Scholar used for supplementary citation tracking and retrieval.

Searches covered the period from 2000 to March 2026. Policy and grey literature were identified in parallel from WHO and World Bank publications, government websites, regional policy documents, and reports from international agencies. Evidence was screened using prespecified eligibility criteria and synthesized using the READ approach for documentary sources [18] and thematic framework analysis across all included materials [19]. Coding followed a hybrid deductive – inductive approach [20], with themes mapped onto the four-quadrant framework to identify facilitators, barriers, and plausible explanatory mechanisms associated with different patterns of UHC progress. Additional details on the search approach, eligibility criteria, coding framework, and country-level evidence mapping are provided in Appendix 2.

## Fixed-effects panel analysis

A separate analytical sample of 26 WPR countries was used for fixed-effects panel modelling after excluding countries with missing covariate data. The panel spanned the period from 2000 to 2022. Fixed-effects panel models were used to examine structural correlates of SDG 3.8.1. This focus was necessitated by the substantially more limited availability of comparable annual data for SDG 3.8.2 [21,22].

Independent variables included GDP per capita, current health expenditure as a percentage of GDP, the proportion of the population aged 65 years and older, mean years of schooling, and the urbanization rate. Covariates were sourced from internationally comparable databases maintained by the World Bank and UN databases. To accommodate the potential for non-linear relationships, GDP per capita was standardized and incorporated into the models as both linear and quadratic terms.

Country fixed effects were incorporated to account for unobserved time-invariant heterogeneity across countries [21,22]. Standard errors were clustered at the country level to address within-country serial correlation and heteroskedasticity [23]. Given the slow-changing nature of several covariates and the limited size of the country sample, the findings were interpreted as structural correlates rather than causal estimates [24]. The implied GDP turning point was reported only when coefficient patterns were consistent with a non-linear association and the estimated value fell within the observed data range [25]. All quantitative analyses were conducted in Stata 18. The model specification was as follows:

$$\mathrm{SDG}3.8.1_{\mathrm{it}}=\beta_{0}+\beta_{1}\mathrm{GD}P_{\mathrm{it}}+\beta_{2}\mathrm{GD}P_{it}^{2}+\beta_{3}\mathrm{HEGD}P_{\mathrm{it}}+\beta_{4}\mathrm{AGE}65_{\mathrm{it}}+\beta_{5}\mathrm{SCHOO}L_{\mathrm{it}}+\beta_{6}\mathrm{URBA}N_{\mathrm{it}}+\alpha_{i}+\varepsilon_{\mathrm{it}}$$

## Integration of quantitative and qualitative evidence

Integration used the four-quadrant typology as a common analytical framework. Quantitative findings were first summarized by quadrant according to country position, temporal movement, and structural correlates of SDG 3.8.1, and qualitative and documentary evidence was then mapped onto the same framework to identify facilitators, barriers, and contextual conditions associated with different profiles [26,27]. Joint displays were used not only to present findings but also to guide cross-quadrant comparison and meta-inference [28]. These displays brought together, for each quadrant, the quantitative profile, broad trajectory pattern, structural correlates of SDG 3.8.1, and the key facilitators and barriers identified from peer-reviewed, policy, and grey literature. This approach enabled the study to move beyond parallel presentation of quantitative and qualitative findings and to generate quadrant-specific interpretations of UHC progress across the WPR.

## Bayesian projection of SDG 3.8 indicators to 2030

Bayesian time-trend models were used as a supplementary forward-looking analysis to project selected SDG 3.8 indicators to 2030 [7,8,11]. Projections were generated for both SDG 3.8.1 and SDG 3.8.2 because annual series were available across the study period. Because the projected indicators were bounded measures on a 0–100 scale, direct modelling on the original scale could lead to heteroscedasticity and predicted values outside the admissible range. Original indicator values were therefore divided by 100 to obtain proportions on the 0–1 scale and were then transformed using the logit function:

$$y_{t}=\log\left(\frac{p_{t}}{1-p_{t}}\right)$$

where $p_{t}$ denotes the proportion form of the indicator in year t, and $y_{t}$ is the transformed continuous outcome. Where necessary, a continuity adjustment was applied to avoid undefined values at the boundaries of 0 or 1. This transformation allowed long-term temporal trends to be modelled on the real line while preserving plausible bounds after back-transformation. A linear time-trend model was specified as:

$$y_{t}=\beta_{0}+\beta_{1}t+\varepsilon_{t},\varepsilon_{t}∼N\left(0,\sigma^{2}\right)$$

where t denotes time, coded with 2000 as the baseline year, $\beta_{0}$ is the intercept, $\beta_{1}$ represents the linear temporal trend, and $\varepsilon_{t}$ is a normally distributed error term with mean 0 and variance $\sigma^{2}$. Models were fitted separately for each indicator series at the regional and country levels, as applicable. A linear specification was used as the main model to preserve comparability across countries and indicators and to characterize long-term trend continuation rather than short-term fluctuation.

Weakly informative priors were specified for model parameters. Regression coefficients were assigned diffuse normal priors:

$$\beta_{0},\beta_{1}∼N\left(0,100\right)$$

and a weakly informative prior was assigned to the residual variance $\sigma^{2}$. Posterior estimation was performed using Markov chain Monte Carlo, with a burn-in of 5000 iterations followed by 20,000 retained samples.

Based on the fitted models, posterior predictive distributions for 2030 were generated as:

$$y_{2030}^{rep}∼N\left(\beta_{0}+\beta_{1}t_{2030},\sigma^{2}\right)$$

Projected values were then back-transformed to the original scale using the inverse logit function:

$$p_{2030}^{\mathrm{pred}}=\mathrm{logi}t^{-1}\left(y_{2030}^{rep}\right)$$

Projected 2030 levels were summarized using posterior predictive means and 95% posterior predictive intervals. These projections were intended to characterize long-term continuation of historical trends under the observed data structure. They were not designed to predict short-term shocks, policy discontinuities, or future changes in financial protection not supported by sufficiently complete country-level data.

## Results

### Regional patterns of SDG 3.8.1 and SDG 3.8.2 in the WPR

**Figure 1.**
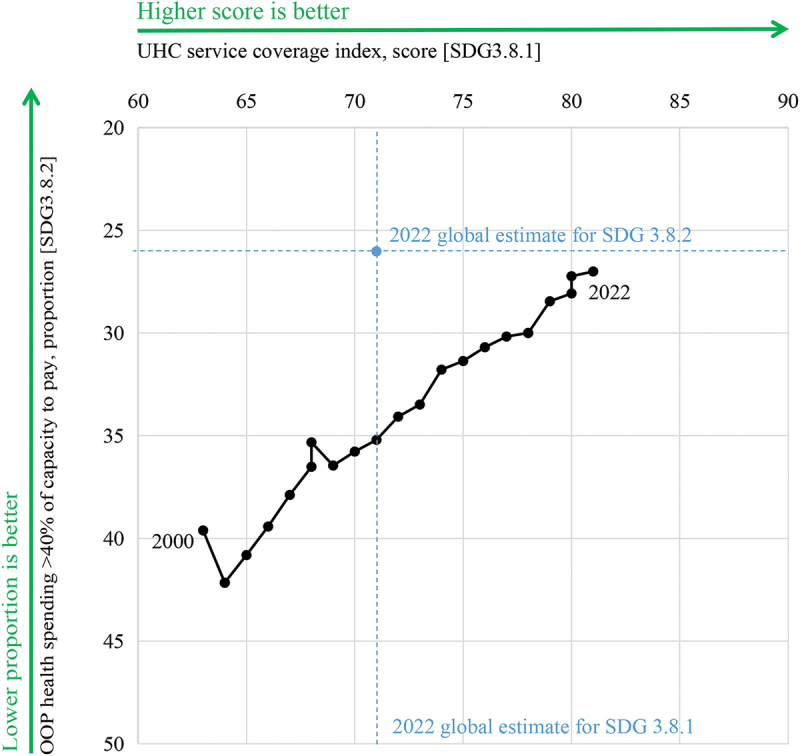
Trends in SDG 3.8.1 and SDG 3.8.2 in the WPR, 2000–2022. Note: SDG 3.8.1 refers to the service coverage index, and SDG 3.8.2 refers to the proportion of the population with out-of-pocket health spending exceeding 40% of household capacity to pay. Data were collected from the WHO Global Health Observatory. The 2022 global estimates for SDG 3.8.1 and SDG 3.8.2 are shown as reference values for comparison.

The WPR demonstrated positive UHC progress, with SDG 3.8.1 increasing from 63 in 2000 to 81 in 2022 and SDG 3.8.2 decreasing from 39.61% in 2000 to 27.00% in 2022. In 2022, the region outperformed the global average on SDG 3.8.1 while its SDG 3.8.2 value remained close to the global level (Figure 1).

**Figure 2.**
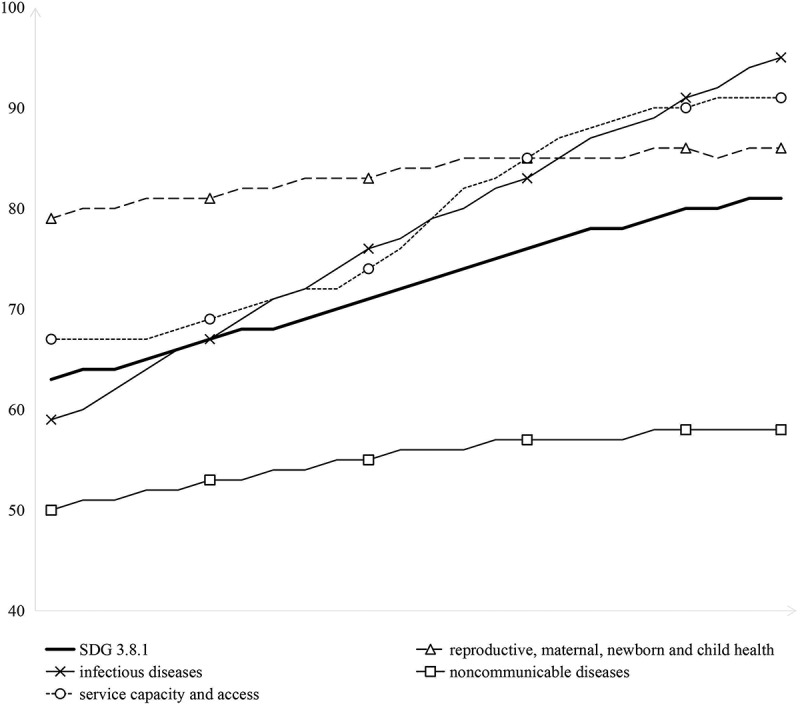
Trends in SDG 3.8.1 and its four sub-indices in the WPR, 2000–2022. Note: SDG 3.8.1 refers to the UHC service coverage index, which is constructed from 14 tracer indicators grouped into four sub-indices: reproductive, maternal, newborn, and child health (RMNCH), infectious diseases, noncommunicable diseases (NCDs), and service capacity and access. Data were extracted from the WHO Global Health Observatory. The index is reported on a 0–100 scale as the geometric mean of these indicators.

Progress across SDG 3.8.1 sub-indices was uneven (Figure 2). RMNCH remained consistently high, infectious diseases and service capacity saw the most substantial improvements, and NCDs lagged with minimal progress, with overall SDG 3.8.1 gains driven by advancements in infectious diseases and service capacity.

**Figure 3.**
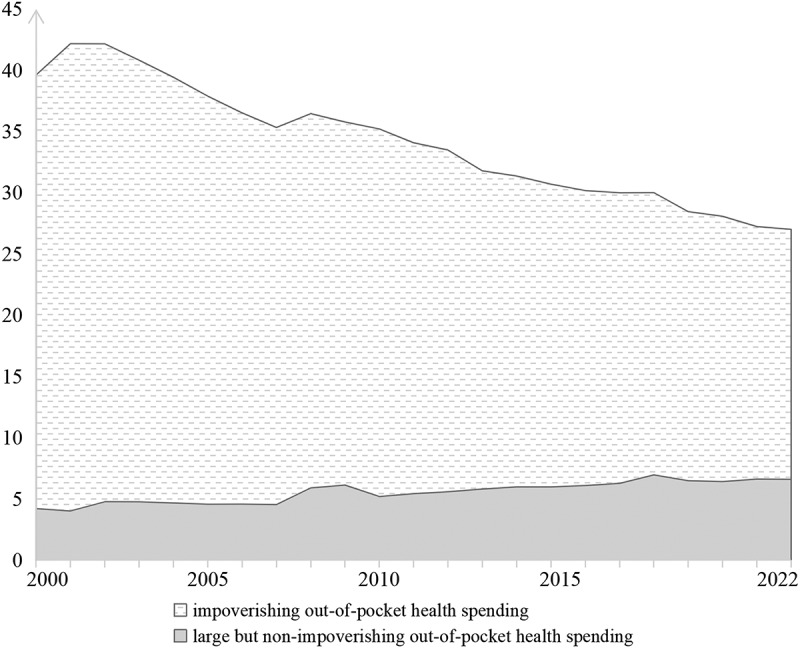
Trends in SDG 3.8.2 and its two sub-indexes in the WPR, 2000–2022. Note: SDG 3.8.2 refers to the proportion of the population with out-of-pocket health spending exceeding 40% of household capacity to pay, including impoverishing out-of-pocket health spending and large but non-impoverishing out-of-pocket health spending. Data were extracted from the WHO Global Health Observatory. Lower values of SDG 3.8.2 indicate less financial hardship.

The decline in SDG 3.8.2 was driven by a steady reduction in impoverishing out-of-pocket health spending, which fell from 35.41% to 20.41%, while large but non-impoverishing out-of-pocket health spending increased modestly from 4.20% to 6.59%, leading to improved overall financial risk protection in the region (Figure 3).

The pace of SDG 3.8.1 improvement slowed after 2015, with an annualized increase of 1.25% between 2000 and 2014 compared to 0.91% between 2015 and 2022, while the annualized decline in SDG 3.8.2 accelerated from −1.67% during the MDG era to −1.83% during the SDG period.

## Four-quadrant distribution and country trajectories

**Figure 4.**
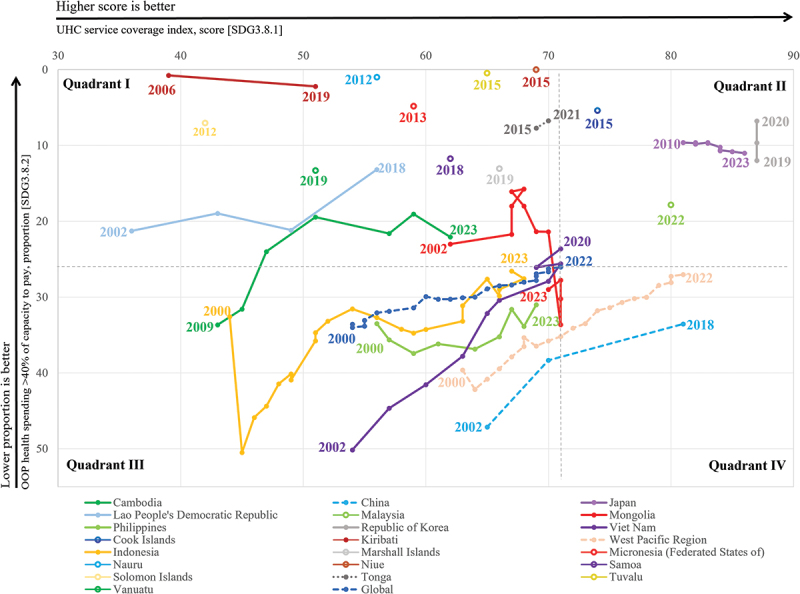
Trends in SDG 3.8.1 and SDG 3.8.2 for 21 countries and areas in the WPR, 2000–2022. Note: SDG 3.8.1 refers to the service coverage index, and SDG 3.8.2 refers to the proportion of the population with out-of-pocket health spending exceeding 40% of household capacity to pay. Data were extracted from the WHO Global Health Observatory. Lower values of SDG 3.8.2 indicate less financial hardship. The 2022 global estimates for SDG 3.8.1 and SDG 3.8.2 are shown as reference values and define the four quadrants used for classification.

Twenty-one countries are then classified into four quadrants based on their joint performance on SDG 3.8.1 and SDG 3.8.2 (Figure 4). Twelve countries, including Cambodia, Kiribati, Lao People’s Democratic Republic, Marshall Islands, Micronesia (Federated States of), Nauru, Niue, Samoa, Solomon Islands, Tonga, Tuvalu, and Vanuatu, were located in the low SDG 3.8.1/low SDG 3.8.2 quadrant. Five countries, including Cook Islands, Japan, Malaysia, Republic of Korea, and Viet Nam, were located in the high SDG 3.8.1/low SDG 3.8.2 quadrant. Three countries, including Indonesia, Mongolia, and the Philippines, were located in the low SDG 3.8.1/high SDG 3.8.2 quadrant. China was the only country located in the high SDG 3.8.1/high SDG 3.8.2 quadrant.

The distribution of historical paired observations was markedly uneven, and largest cluster was in the low SDG 3.8.1 and low SDG 3.8.2 quadrant (Appendix 3). This group was dominated by Pacific Island countries and territories (PICTs), which accounted for 10 of the 12 countries in the quadrant. Data availability was also limited in this group, as most of these island settings had only one paired observation. Only Kiribati and Tonga had more than one paired observation, and both showed modest improvement over time. Cambodia and Lao People’s Democratic Republic, the two non-island countries in this quadrant, also recorded gains in SDG 3.8.1 and reductions in SDG 3.8.2, but remained below the more favorable configurations shown in the figure.

The high SDG 3.8.1 and low SDG 3.8.2 quadrant represented the most favorable joint configuration, but it included only five countries. Japan and the Republic of Korea showed the strongest and most stable profiles, combining high service coverage with persistently low financial hardship. By contrast, Cook Islands and Malaysia were represented by only one paired observation each. Viet Nam showed a more dynamic trajectory, with substantial improvement in SDG 3.8.1 accompanied by a marked decline in SDG 3.8.2, making it one of the clearest examples of combined progress in both dimensions.

The low SDG 3.8.1 and high SDG 3.8.2 quadrant included Indonesia, Mongolia, and the Philippines. These countries were characterized by incomplete service coverage and persistently high financial hardship. Although all three countries recorded some improvement in SDG 3.8.1 over time, progress in SDG 3.8.2 was weaker and more unstable than in the more favorable quadrants. What’s more, the high SDG 3.8.1 and high SDG 3.8.2 quadrant was occupied only by China. This profile indicated that strong service coverage could coexist with a persistently high level of financial hardship.

## Country trajectories and cross-quadrant movement

Three broad trajectory patterns were identified. The first was a stable favorable pattern, most clearly represented by Japan and the Republic of Korea. Japan maintained SDG 3.8.1 above 80 throughout the observation period, increasing from 81 to 86, while SDG 3.8.2 remained low at around 9.6% to 11%. The Republic of Korea showed a similarly stable profile. These two countries, therefore, represented the most balanced UHC profiles in the region.

The second pattern was gradual but incomplete improvement. Cambodia and Lao People’s Democratic Republic both showed sustained increases in SDG 3.8.1 alongside overall declines in SDG 3.8.2, although neither reached the more favorable joint configuration. Viet Nam recorded one of the strongest combined improvements, with SDG 3.8.1 rising from 54 to 71 and SDG 3.8.2 falling from 50.15% to 23.61%. Indonesia also improved steadily in SDG 3.8.1, from 44 in 2000 to 67 in 2023, but progress in SDG 3.8.2 was smaller and less stable. Overall, these trajectories suggest that gains in service coverage were often more sustained than gains in financial protection.

The third pattern was fragmented or stalled progress. Mongolia showed only modest improvement in SDG 3.8.1, while SDG 3.8.2 worsened overall and peaked in 2020. The Philippines also improved in SDG 3.8.1, but SDG 3.8.2 remained persistently high throughout the period. China showed a distinct pattern of partial convergence: SDG 3.8.1 rose markedly, but SDG 3.8.2, although declining, remained substantially higher than in the countries with high SDG 3.8.1 and low SDG 3.8.2. This indicates that rapid expansion of service coverage did not necessarily translate into equally strong improvements in financial protection.

## Structural correlates of the service-coverage dimension

A country fixed-effects panel model was estimated using 598 country-year observations from 26 countries in the Western Pacific Region over 2000–2022 to examine structural correlates of SDG 3.8.1 (Table 1).

**Table 1.** Fixed-effects panel regression analysis on determinants of SDG 3.8.1 in the WPR, 2000–2022.

SDG3.8.1	Coefficient	Std. Err.	p-Value	95% CI
Std. GDP per capita	5.975324***	1.987789	0.006	[1.881395, 10.06925]
Std. GDP per capita (squared)	−1.643234***	0.5338198	0.005	[−2.742656, −0.5438115]
Health expenditure (% of GDP)	−0.3077792	0.2022438	0.141	[−0.7243081, 0.1087497]
Population aged 65+ (% of total)	0.5180113	0.3554123	0.157	[−0.213974,1.249997]
Average years of schooling	2.489796**	0.9758656	0.017	[0.47999628,4.499629]
Urbanization rate (%)	0.4471927**	0.1645769	0.012	[0.1082402, 0.7861453]
cons	17.65388	10.63578	0.109	[−4.250925, 39.55869]

Notes: Coefficients are reported with country-clustered robust standard errors in parentheses. Significance levels: *p < 0.10, **p < 0.05, ***p < 0.01. The analysis uses a 26-country panel covering 2000–2022. The dependent variable is SDG 3.8.1. ‘Std. GDP per capita’ refers to z-score standardized GDP per capita, and ‘Std. GDP per capita (squared)’ refers to its squared term, included to test the inverted U-shaped relationship between GDP per capita and SDG 3.8.1. ‘cons’ denotes the constant term.

The sample consists of 598 country-year observations from 26 countries spanning the period from 2000 to 2022. The results reveal a significant U-shaped relationship between GDP per capita and SDG 3.8.1. The coefficient for standardized GDP per capita is positive and statistically significant (*p* < 0.01), indicating that as GDP per capita increases, SDG 3.8.1 improves. However, the squared term of standardized GDP per capita has a significant negative effect (coefficient = −1.643234, *p* < 0.01), which suggests a concave curve, indicating that the positive impact of GDP per capita on SDG 3.8.1 diminishes at higher levels of GDP. This U-shaped relationship implies that while increases in GDP per capita contribute positively to UHC, the effect diminishes and eventually reverses at a certain threshold. The turning point of this relationship, where the effect shifts from positive to negative, is calculated at 40,146.14 USD. This value represents the GDP per capita at which the marginal effect of GDP on SDG 3.8.1 reaches its peak before beginning to decline.

Average years of schooling has a significant and positive impact on SDG 3.8.1, underscoring the importance of education in improving health coverage. The urbanization rate also shows a positive and statistically significant effect, suggesting that urbanization contributes to enhanced UHC. By contrast, health expenditure as a percentage of GDP is not statistically significant, indicating no direct association with SDG 3.8.1 in this context, and the proportion of the population aged above 65 years old shows a positive but insignificant effect.

## Integrated facilitators and barriers across quadrant profiles

Integrated analysis of quadrant profiles, country trajectories, and documentary evidence identified two broad coding domains: health-system factors, structured around the WHO six building blocks, and broader social determinants (Table 2). Across quadrants, UHC progress was most consistently supported by sustained political commitment, expanded pooled public financing, stronger primary health care orientation, improved workforce and service-delivery capacity, and more coherent governance and health information systems. Recurrent barriers included fragmented financing, persistent out-of-pocket payments, workforce shortages and maldistribution, weak service readiness, geographic remoteness, infrastructure deficits, climate vulnerability, and population ageing.

**Table 2.** Health-system factors and broader social determinants associated with different patterns of UHC progress.

Quadrant	Countries	Health-system factors	Broader social determinants
Quadrant I low SCI/low FH	Cambodia Kiribati Lao PDR Marshall Islands Micronesia (Federated States of) Nauru Niue Samoa Solomon Islands Tonga Tuvalu Vanuatu	Facilitators: Pooled insurance reform Sin tax Barriers: Heavily rely on international aid program Constrained access to fundamental services Imbalanced distribution and persistent migration of skilled healthcare workers Insufficient digital health services	Facilitators: Economic growth Barriers: Escalating climate and disaster shocks Geographic dispersion Private sector’s growth
Quadrant II high SCI/low FH	Cook Islands Japan Malaysia Republic of Korea Viet Nam	Facilitators: Pooled insurance reforms Strong political commitment to primary health care Effective health information systems	Facilitators: Economic growth Barriers: Aging population
Quadrant III low SCI/high FH	Indonesia Mongolia Philippines	Facilitators: Pooled insurance reform Government’s commitment to decentralization Sin tax Barriers: Low government health expenditure Private sector’s growth Unequal distribution of healthcare workers Limited capacity of public health care services Low coverage of social health protection schemes Fragmentation between public and private services	Barriers: Income inequity and poverty Geographic dispersion Cultural and social factors (nomadic lifestyle)
Quadrant IV high SCI/high FH	China	Facilitators: Continued and strong political commitment Investment in primary health care National health insurance system Barriers: Escalating healthcare expenses Large share of OOP payment	Facilitators: Economic growth Barriers: Aging population

Notes: This table summarizes facilitators and barriers identified through the explanatory evidence review and structured document analysis. Factors were mapped to the four-quadrant framework to support interpretation of different patterns of UHC progress. Quadrant classification was based on the 2022 global estimates of SDG 3.8.1 and SDG 3.8.2 used as reference values.

Within the health-system domain, political commitment and governance coherence emerged as common facilitators in countries with more favorable or improving profiles. National initiatives like the Healthy China 2030 Blueprint and consistent centering UHC in the national healthcare reform since 2009 have set ambitious goals for the country [29,30]. In Viet Nam, subsidized insurance expansion for poor and vulnerable groups supported continued gains in service coverage and financial protection [31–33]. In the Cook Islands, Te Marae Ora provided central stewardship and linked health planning to the National Sustainable Development Plan [34,35]. Across these settings, documentary evidence consistently linked long-term policy commitment with more sustained UHC progress.

A second cross-quadrant facilitator was pooled public financing and insurance reform. Cambodia expanded the Health Equity Fund and the National Social Security Fund for Healthcare, increasing population coverage from 23.5% in 2015 to 43.7% in 2024, alongside a 114% increase in health expenditure and a greater shift towards domestic public financing [36]. Lao People’s Democratic Republic moved from fragmented risk-sharing arrangements to a National Health Insurance scheme covering 94.5% of the population [37]. In Lao People’s Democratic Republic, the Tobacco Control Fund and later excise reforms also created additional fiscal space for health [38]. Taken together, these findings indicate that broader pooling and more stable public financing were repeatedly associated with more favorable UHC trajectories.

A third facilitator was stronger PHC, service-delivery organization, and digital or information capacity. Japan’s medical Digital Transformation(DX) strategy expanded telemedicine, mobile health applications, and real-time data systems, with electronic medical record adoption expected to exceed 90% by 2024 [39]. Viet Nam used digital outreach, including virtual HIV self-testing platforms, to reach underserved groups [40]. China strengthened PHC and rural general practitioner training after 2009 [29,41]. Across these examples, service coverage improved most where financing expansion was accompanied by stronger delivery platforms and better continuity of care.

The most recurrent barrier across quadrants was incomplete financial protection despite coverage expansion. In Cambodia, the large role of private and informal providers expanded access but also sustained high OOP payments and uneven quality. In China, universal insurance coverage coexisted with high deductibles, persistent co-payments, and low outpatient reimbursement [31,42]. In Indonesia, Mongolia, and the Philippines, limited government health expenditure, fragmented provider arrangements, and incomplete social protection continued to weaken financial risk protection. These patterns suggest that insurance breadth did not necessarily translate into insurance depth.

A second major barrier was weak workforce and service capacity. In Cambodia, workforce density in 2024 remained at 7.3 doctors and 15.7 nurses per 10,000 population, below regional low- and lower-middle-income averages. Lao People’s Democratic Republic reported a workforce density of 1.23 per 1,000 population in 2022, well below both the UHC benchmark of 4.45 and the national target of 1.7 [43]. Mongolia’s PHC sector faced limited diagnostic capacity and shortages of essential medicines [44]. In many settings, these shortages were compounded by weak digital infrastructure, poor referral coordination, and marked rural – urban disparities.

Within the broader social determinants’ domain, geographic remoteness, weak infrastructure, climate shocks, and ageing were recurrent barriers. In Kiribati, 76% of the population lacked access to clean water and sanitation, while in Solomon Islands the comparable figure was 67% [45]. Reliable sanitation remained limited in Kiribati (45%), Solomon Islands (35%), and Vanuatu (51%), and open defecation reached 45% in Solomon Islands [46]. Climate-related threats further intensified these constraints in Pacific settings [47,48]. Population ageing also emerged as a cross-quadrant challenge. In Japan, households with older members were more likely to incur catastrophic health expenditure [49]. In the Republic of Korea, total health spending reached USD 135 billion in 2021, equivalent to 9.3% of GDP, while persistent OOP payments continued to expose older households to financial hardship [50]. Similar concerns were reported for China [51].

## Quadrant-specific contextual mechanisms

Although these factors recurred across the region, their configuration differed by quadrant profile.

In the low SDG 3.8.1 and low SDG 3.8.2 quadrant, the integrated evidence suggested two distinct mechanisms. In Cambodia and Lao People’s Democratic Republic, this profile reflected gradual but incomplete UHC progress, supported by pooled financing reform, stronger domestic investment, and consolidation of insurance arrangements [37]. In many PICTs, however, the same profile more often reflected limited service availability, sparse financial hardship data, and financing arrangements that shifted high-cost care from households to governments through overseas referral systems [47,52–54]. In these settings, low measured financial hardship should be interpreted cautiously rather than as evidence of secure financial protection. Complementary evidence also pointed to persistent constraints in workforce supply [43], digital readiness [44], water and sanitation [45,46], climate resilience [48], and dependence on external aid [47].

In the high SDG 3.8.1 and low SDG 3.8.2 quadrant, more favorable performance was associated with coherent governance, stronger risk pooling, broader primary health care platforms, and better information systems. Japan and the Republic of Korea represented relatively mature systems that sustained high service coverage with low financial hardship. Viet Nam approached this profile through subsidized insurance expansion, tax-financed programmes, and a broad public provider network from commune to central level [31,32]. The Cook Islands likewise suggested the importance of central stewardship and integrated service organization in a small-island setting [34,35,52]. The main constraint in this quadrant was population ageing, which increased demand and expenditure pressure even where service coverage was already high [49,50,55].

In the low SDG 3.8.1 and high SDG 3.8.2 quadrant, represented by Indonesia, Mongolia, and the Philippines, service coverage remained incomplete and financial hardship remained high. Documentary evidence linked this pattern to low public health expenditure, incomplete social protection, fragmented provision, weak service readiness, and difficult geography. In Indonesia, decentralization and primary care strengthening created a basis for future improvement [55], but fragmentation and private-sector expansion continued to constrain progress. In Mongolia, geographic dispersion and nomadic livelihoods further limited continuity of care [56]. In this quadrant, disadvantages tended to accumulate rather than occur in isolation.

In the high SDG 3.8.1 and high SDG 3.8.2 quadrant, represented only by China, service expansion clearly outpaced improvement in financial protection. China achieved universal insurance coverage by 2011 and made substantial gains in service coverage [57]. These gains were supported by strong political commitment, workforce expansion, and primary health care investment [29,30,41]. However, high deductibles, persistent co-payments, and limited outpatient reimbursement continued to weaken financial protection [31,58]. This profile therefore reflected broad insurance coverage without equally strong depth of protection. Rapid population ageing further intensified this pressure [51].

## Projected trajectories of SDG 3.8 indicators to 2030

Consistent with the historical trajectories and the integrated analysis of facilitators and barriers, Bayesian projections suggested that UHC in the WPR will continue to improve through 2030 in both service coverage and financial protection, although progress in service coverage is likely to remain uneven across domains (Table 3). At the regional level, the overall SDG 3.8.1 index was projected to rise from 81.50 in 2022 to 85.62 in 2030, while SDG 3.8.2 was projected to decline from 26.13 to 22.16, indicating continued improvement in financial protection under the observed historical trend structure.

**Table 3.** Observed and projected SDG 3.8.1, its sub-indices, and SDG 3.8.2 in the WPR, 2022 and 2030.

Indicator	2000	2010	2015	2022	2030
SDG 3.8.1	62.06 (59.98–64.10)	71.56 (69.83–73.21)	75.73 (74.15–77.22)	81.50 (80.14–82.79)	85.62 (84.44–86.74)
RMNCH	79.87 (78.01–81.61)	83.08 (81.53–84.51)	84.52 (83.05–85.86)	86.62 (85.26–87.87)	88.25 (86.96–89.44)
Infectious diseases	54.51 (46.68–62.12)	77.34 (71.68–82.11)	85.21 (80.97–88.58)	93.01 (90.67–94.80)	96.51 (95.21–97.49)
NCDs	51.04 (48.85–53.24)	54.61 (52.52–56.66)	56.37 (54.27–58.41)	59.16 (57.01–61.28)	61.55 (59.32–63.76)
Service capacity and access	59.80 (50.84–68.16)	77.06 (70.38–82.57)	83.47 (78.04–87.70)	90.63 (87.06–93.32)	94.47 (92.09–96.20)
SDG 3.8.2	41.93 (39.29–44.63)	34.61 (32.28–37.00)	31.19 (28.97–33.45)	26.13 (24.03–28.31)	22.16 (20.14–24.31)

Notes: Observed values refer to 2022 regional estimates for the WPR, and projected values refer to 2030 estimates. Projections were generated using Bayesian linear time-trend models fitted to historical annual series. Values in parentheses indicate 95% posterior predictive intervals for the projected estimates. Because the indicators are bounded on a 0–100 scale, values were rescaled to the 0–1 interval and logit-transformed before modelling, and projected values were back-transformed to the original scale for presentation.

Projections of the four SDG 3.8.1 sub-indices showed clear divergence in both level and pace of improvement. The infectious diseases subdimension was projected to remain the strongest-performing component, increasing from 93.01 in 2022 to 96.51 in 2030. Service capacity and access was also projected to remain high, rising from 90.63 to 94.47. RMNCH, already at a comparatively high level, was projected to improve more modestly, from 86.62 to 88.25, suggesting relative stabilization at an advanced level. By contrast, the NCD subdimension remained persistently the weakest throughout the projection period, increasing only from 59.16 to 61.55.

These projections indicate the WPR will likely maintain an overall positive UHC trajectory through 2030, with continued gains in both service coverage and financial protection. However, the projected improvement in service coverage remains clearly imbalanced. Future gains in the overall SDG 3.8.1 index are expected to be driven mainly by progress in already strong domains, particularly infectious diseases and service capacity and access, while NCD-related service coverage is likely to remain the main bottleneck. Even by 2030, the projected NCDs subindex will be more than 34 points lower than that of infectious diseases and nearly 33 points lower than service capacity and access.

## Discussion

Overall, UHC in the WPR improved substantially, but progress remained uneven across countries and across SDG 3.8.1 and SDG 3.8.2. Gains in SDG 3.8.1 were not always matched by comparable improvements in SDG 3.8.2, indicating that service expansion often outpaced financial protection. The NCD sub-index remained the weakest part of service coverage throughout the study period and is likely to remain so by 2030.

Existing studies have substantially advanced UHC measurement and policy analysis, but several important gaps remain. Much of the literature has focused on single-country analyses [7,8], on only one dimension of UHC [9], or on context-specific adaptations of monitoring frameworks [10,11], with less attention to how service coverage and financial protection evolve together across countries and over time, particularly in highly heterogeneous regions such as the WPR. In addition, prior work has often relied either on descriptive monitoring or on country- and policy-specific case studies [3], while fewer studies have jointly integrated service coverage, financial hardship, trajectories, and contextual explanation within a common analytical framework [59,60].

Therefore, this study extends the literature in several respects. First, it jointly examines SDG 3.8.1 and SDG 3.8.2, in line with current global UHC monitoring that treats them as complementary indicators. Second, it uses a four-quadrant framework to identify cross-country mismatches and trajectories in service coverage and financial protection. Third, it integrates panel analysis with qualitative and documentary evidence to interpret the mechanisms underlying different country patterns. Finally, by focusing on the WPR as a comparative regional setting, it reveals forms of uneven UHC progress that are less apparent in single-country studies, including the persistent weakness of the NCD service dimension. Overall, this approach moves beyond single-dimension or single-country assessments and provides a more policy-relevant account of uneven UHC progress in the region.

A central finding of this study is that gains in service coverage did not always translate into equally strong improvements in financial protection. This mismatch was particularly evident in the WPR, where countries could occupy similar positions in one UHC dimension while differing substantially in the other. In many PICTs, both SDG 3.8.1 and SDG 3.8.2 remained low, but the interpretation of this pattern requires caution. On the one hand, low SDG 3.8.1 reflects persistently limited-service availability, weak primary health care (PHC) readiness, and substantial unmet need, particularly outside urban centers. On the other hand, a low level of financial hardship does not necessarily imply strong financial protection. In these settings, low SDG 3.8.2 may partly reflect underutilization of services, single-year data availability, and the role of publicly financed overseas referral arrangements, which shift part of the cost burden away from direct household out-of-pocket payments and may mechanically suppress reported financial hardship. Accordingly, low financial hardship in such contexts should not be interpreted in isolation from low service coverage, because low utilization itself may conceal unmet need rather than genuine protection.

This divergence between the two UHC dimensions was also visible in countries where service coverage expanded more rapidly than financial protection. China illustrates this pattern clearly. Although service utilization and insurance coverage expanded substantially, achieving over 95% population insurance coverage as early as 2013 [42], improvements in financial protection were more limited, suggesting that insurance breadth did not necessarily translate into comparable depth of protection. When deductibles, co-payments, and gaps in outpatient reimbursement remain substantial, expanded service use may coincide with persistently high household spending. In such settings, rising SDG 3.8.1 can coexist with only modest reductions in SDG 3.8.2, or even with increased financial pressure if public financing does not keep pace with utilization growth. This helps explain why some countries moved towards higher service coverage without achieving equally favorable financial protection outcomes. Taken together, these findings suggest that SDG 3.8.1 and SDG 3.8.2 should be interpreted as related but not interchangeable indicators of UHC progress: expansion in coverage is necessary, but without adequate pooling, benefit design, and cost protection, it may not be sufficient to reduce financial hardship.

Within this broader UHC framework, strengthening PHC appears to be one of the most important mechanisms for improving both service coverage and financial protection. PHC expands access to essential services at community level, improves continuity of care, and reduces reliance on delayed or high-cost hospital-based treatment [61,62]. Quantitative evidence indicates that each 1% increase in PHC expenditure per capita is associated with an approximate 0.14-point improvement in UHC index scores – an effect that is statistically significant though inelastic [63]. Evidence from Vietnam and other settings suggests that even under constrained fiscal conditions, stronger PHC systems, community engagement, and local delivery capacity can improve access and prevention while also helping to moderate the downstream financial burden associated with untreated disease or avoidable complications [64]. This is particularly relevant for the WPR, where the persistent weakness of the NCD sub-index indicates that many health systems have expanded coverage more successfully for infectious diseases and maternal and child health than for chronic disease prevention and long-term management. In this sense, PHC is not only a delivery platform but also a policy bridge between the two dimensions of UHC.

The findings also underscore that financing arrangements and governance structures shape whether gains in service coverage are converted into effective financial protection. More favorable quadrant patterns were commonly associated with pooled public financing, stronger governance, better service-delivery capacity, and more coherent health information systems, whereas less favorable profiles were more often marked by fragmented financing, persistent out-of-pocket payments, workforce shortages, and weak service readiness [1]. The private sector may contribute additional resources and service capacity, especially where public provision is limited, but its role is double-edged. Without effective stewardship, private provision may reinforce urban bias, widen geographic inequalities, and expose households to higher direct payments, thereby weakening the financial protection dimension of UHC even where service availability expands. Experience from Cambodia illustrates this risk, where strong reliance on private providers has been associated with uneven access and continued household financial burden [65]. The key issue, therefore, is not simply whether services are available, but whether they are organized, financed, and regulated in ways that allow increased utilization to occur without deepening household financial burden.

Finally, the relationship between service coverage and financial protection must also be understood in light of broader structural constraints. In the PICTs, geographic remoteness and climate-related disruption can affect both dimensions simultaneously by limiting service availability while increasing the cost and complexity of delivery. Population ageing presents a different but equally important challenge across the region. As the burden of chronic disease rises, health systems face increasing demand for long-term management, medicines, and outpatient care, all of which place pressure on both service capacity and social protection mechanisms. Without sustained investment in PHC, workforce, financing reform, and chronic care delivery, progress in SDG 3.8.1 may continue to outpace improvements in SDG 3.8.2. This reinforces the importance of monitoring the two indicators together, particularly in regions where UHC progress remains uneven and where the weakest dimension may become the main constraint on further gains.

## Strengths and limitations

By jointly examining SDG 3.8.1 and 3.8.2, it assessed UHC progress across service coverage and financial protection. The four-quadrant framework offered a clear comparative structure to identify cross-country mismatches and trajectories in the WPR, while the mixed-methods design – combining descriptive, panel, qualitative, documentary, and projection analyses – supported both pattern identification and contextual interpretation. Focusing on the heterogeneous WPR highlighted uneven progress, including the persistent weakness of NCD services.

Several limitations should be noted. Reliance on WHO-reported SDG indicators allows international comparability but may not fully capture country-specific needs, quality, or subnational disparities. The four-quadrant framework is descriptive and may simplify complex realities, and the qualitative component depended on published sources rather than primary field data. Fixed-effects models identify structural correlates of SDG 3.8.1 but do not establish causality, and residual confounding may remain. Limited SDG 3.8.2 data constrained full two-dimensional panel analysis, and Bayesian projections reflect historical trend continuation rather than abrupt policy or economic changes, requiring cautious interpretation.

## Conclusions

First of all, most PICTs are stuck in a situation where service coverage remains low and financial protection is weak, so progress needs to move beyond nominal expansion toward systematically addressing unmet need. Secondly, strengthening PHC should be the core of this shift, expanding equitable community-based access while improving system performance and coordination. Thirdly, the private sector can contribute capacity and responsiveness, but it needs clear stewardship to avoid fragmentation, rising costs, and unequal care. Lastly, digital health, tailored to local needs, can also help overcome distance and workforce constraints and improve continuity of care, especially for remote and underserved populations. Overall, durable gains will depend on aligning primary health care focused reform, accountable governance of public and private provision, scalable innovation, and multisectoral action in the face of growing climate and demographic pressures.

## Supplementary Material

supplementary file clean version.docx

## Data Availability

The datasets analyzed during the current study are publicly available from the sources described in the Methods section and cited in the references.
