# Supplementary material for: Four-quadrant analysis of universal health coverage progress in the Western Pacific region: facilitators, barriers, and trajectories to 2030
Source: Glob Health Action. 2026 Apr 21;19(1):2659459. doi: 10.1080/16549716.2026.2659459 (PMC13101004; doi:10.1080/16549716.2026.2659459)

**Supplementary Appendix 1: variables**

| Variable | Definition | Source |
| --- | --- | --- |
| **SDG 3.8.1** | UHC service coverage index based on 14 tracer indicators across reproductive, maternal, newborn, and child health (RMNCH), infectious diseases, noncommunicable diseases (NCDs), and service capacity and access | WHO Global Health Observatory |
| **SDG 3.8.2** | Proportion of the population incurring out-of-pocket health spending exceeding 40% of household capacity to pay | WHO Global Health Observatory |
| **GDP per capita** | Gross domestic product divided by mid-year population, expressed in current US$ | World Bank |
| **Current health expenditure (% of GDP)** | Current health expenditure expressed as a share of gross domestic product | World Bank / WHO Global Health Expenditure Database |
| **Population aged 65+ (%)** | Population aged 65 years and above as a percentage of the total population | World Bank / UN World Population Prospects |
| **Mean years of schooling** | Average number of years of education received by adults aged 25 years and older | UNDP Human Development Report Office |
| **Urbanization rate (%)** | Urban population as a percentage of the total population | World Bank / UN World Urbanization Prospects |

**Supplementary Appendix 2. Additional details for the explanatory evidence review and structured document analysis**

**Supplementary Table 2.1. Search sources and search domains**

| Source | Coverage | Purpose |
| --- | --- | --- |
| **PubMed** | **Peer-reviewed literature** | **UHC measurement, determinants, and policy pathways** |
| **Web of Science** | **Peer-reviewed literature** | **Broader policy and comparative health systems studies** |
| **Scopus** | **Peer-reviewed literature** | **Additional international and regional studies** |
| **Google Scholar** | **Supplementary search** | **Citation tracking and retrieval of additional relevant studies** |
| **WHO/WPRO, World Bank, government and ministry websites, regional policy documents, and reports from international agencies** | **Policy and grey literature** | **Documentary and policy evidence relevant to country- and quadrant-level interpretation** |

Notes: Search strings were adapted to the syntax and indexing conventions of individual databases and sources. Representative search concepts included “universal health coverage”, “service coverage”, “financial hardship”, “health financing”, “primary health care”, “governance”, “workforce”, “Western Pacific”, “Pacific Islands” et al, and relevant country names.

**Supplementary Table 2.2. Eligibility criteria for peer-reviewed and documentary sources**

| Domain | Included | Excluded |
| --- | --- | --- |
| Topic relevance | Sources directly addressing UHC measurement, service coverage, financial protection, financial hardship, catastrophic health expenditure, health-system determinants, policy pathways, facilitators, or barriers | Sources with no substantive relevance to UHC or health-system performance |
| Geographic relevance | WPR-focused sources; Pacific Island countries and territories; Asia-Pacific or comparable settings where findings were conceptually relevant | Sources with no relevance to the WPR or no clear conceptual relevance to the study framework |
| Type of evidence | Empirical studies, comparative analyses, policy reports, monitoring reports, strategic plans, health financing assessments, service delivery profiles, and technical reports | Pure commentary, brief opinion pieces, news items, promotional material, or sources without substantive analytical content |
| Policy and grey literature | WHO/WPRO, World Bank, UN documents, government publications, regional strategies, and attributable technical reports | Non-attributable, non-verifiable, or purely promotional materials |
| Clinical focus | Studies linking disease-specific issues to UHC, PHC, service delivery, or financial protection | Studies focused solely on clinical efficacy or treatment outcomes without connection to UHC or system performance |
| Time period | Published or issued between 2000 and March 2026 | Outside this range unless exceptionally foundational and explicitly justified |

## **Analytical approach**

Included materials were reviewed using a structured analytical matrix that recorded source type, geographic focus, UHC dimension, main topic, key findings, interpretation, and quadrant relevance. Initial thematic categories were informed by the study framework, while additional themes were generated inductively during review of country-specific materials. Themes were subsequently classified as facilitators, barriers, or mixed/context-dependent influences and mapped to the four-quadrant framework to support interpretation of country positioning and movement over time.

Evidence was interpreted as convergent when documentary or literature-based explanations aligned with the quantitative profile, complementary when they added context to the observed pattern, and discordant when they suggested a different or more complex interpretation requiring caution.

**Supplementary Table 2.3. Analytical coding framework**

| Overarching domain | Thematic category | Example subthemes | Relevance to quadrant interpretation |
| --- | --- | --- | --- |
| Health-system factors | Health financing and pooling | Health insurance schemes; medical service cost; current health expenditure; dependence on external aid; financial risk protection; private sector not covered by social health insurance; other financing issues | Helps explain variation in both SDG 3.8.1 and SDG 3.8.2 |
| Health-system factors | Benefit design and financial protection | Copayments; deductibles; reimbursement depth; outpatient coverage; exclusions from benefit packages | Helps interpret mismatch between service coverage and financial protection |
| Health-system factors | Primary health care orientation | Strength of PHC; gatekeeping; continuity of care; community-based service organisation | Relevant to service coverage performance |
| Health-system factors | Human resources for health | Workforce size and skill mix; workforce distribution; brain drain; dependence on overseas health workers; training capacity; incentives and motivation | Relevant to service availability, continuity, and rural access |
| Health-system factors | Service delivery and readiness | Organisation of service delivery; PHC capacity; maternal health capacity; access in rural and remote areas or small islands; infrastructure; service quality; referral arrangements | Strongly linked to service coverage trajectories |
| Health-system factors | Health information systems | HIS development; digital technology adoption; investment in HIS; data integration; monitoring capacity | Relevant to implementation capacity and system coordination |
| Health-system factors | Medicines, vaccines, and technology | Access to medicines, vaccines, and technology; maldistribution; reimbursement policy; generic substitution; diagnostic capacity | Relevant to effective coverage and affordability |
| Health-system factors | Governance and implementation | Separation of provision and purchasing; decentralisation; political commitment; reform continuity; stewardship and coordination | Relevant to sustained UHC progress |
| Broader contextual determinants | Economic context | Economic growth; fiscal capacity; economic stability; income inequality; poverty reduction; other economic context issues | Shapes fiscal space, affordability, and sustainability |
| Broader contextual determinants | Geography | Small-island settings; remote areas; transport barriers; dispersed populations | Particularly relevant to Pacific Island and remote-country settings |
| Broader contextual determinants | Demographic context | Population size; ageing; internal and international migration; other population issues | Influences service demand and long-term system pressure |
| Broader contextual determinants | Climate change | Climate-related disruptions and vulnerability | May constrain service delivery and fiscal resilience |
| Broader contextual determinants | Political instability | Instability, discontinuity, governance fragility | Relevant to stalled or unstable UHC progress |
| Broader contextual determinants | Other context issues | Country-specific enabling conditions or constraints not captured above | Used where additional cross-cutting themes emerged inductively |

Note: Thematic categories could be coded as facilitators, barriers, or mixed/context-dependent influences depending on their direction and role in the country-specific interpretation.

**Supplementary Table 2.4. Analytical matrix template used for country-level evidence mapping of facilitators and barriers related to UHC progress in the WPR**

|  | Australia | Brunei Darussalam | Cambodia | China | Cook Islands | … | Viet Nam |
| --- | --- | --- | --- | --- | --- | --- | --- |
| 1.1 Health insurance scheme |  |  |  |  |  |  |  |
| 1.2 Medical service cost |  |  |  |  |  |  |  |
| 1.3 Current health expenditure (as % of GDP) |  |  |  |  |  |  |  |
| 1.4 Dependence on external aid |  |  |  |  |  |  |  |
| 1.5 Financial risk protection |  |  |  |  |  |  |  |
| 1.6 Large private sector not covered by SHI |  |  |  |  |  |  |  |
| 1.7 Other health financing factors |  |  |  |  |  |  |  |
| 2.1 Healthcare workforce size and skill |  |  |  |  |  |  |  |
| 2.2 Distribution of the health workforce |  |  |  |  |  |  |  |
| 2.4 Brain drain (emigration) |  |  |  |  |  |  |  |
| 2.5 Dependence on overseas health workers |  |  |  |  |  |  |  |
| 2.6 Medical education/training capacity |  |  |  |  |  |  |  |
| 2.7 Incentives and motivation |  |  |  |  |  |  |  |
| 2.8 Other HRH issues |  |  |  |  |  |  |  |
| 3.1 Organization |  |  |  |  |  |  |  |
| 3.2 PHC capacity |  |  |  |  |  |  |  |
| 3.3 Maternal health capacity |  |  |  |  |  |  |  |
| 3.4 Access in rural and remote areas/small islands |  |  |  |  |  |  |  |
| 3.5 Private sector |  |  |  |  |  |  |  |
| 3.6 Overuse of inpatient services |  |  |  |  |  |  |  |
| 3.6 Dependence on overseas referral service |  |  |  |  |  |  |  |
| 3.7 Infrastructure |  |  |  |  |  |  |  |
| 3.8 Service quality |  |  |  |  |  |  |  |
| 3.9 Other service delivery issues |  |  |  |  |  |  |  |
| 4.1 Level of HIS development and application of digital technology |  |  |  |  |  |  |  |
| 4.2 Level of investment in HIS |  |  |  |  |  |  |  |
| 4.3 Other HIS issues |  |  |  |  |  |  |  |
| 5.1 Access to Medicine/Vaccine/Technology |  |  |  |  |  |  |  |
| 5.2 Maldistribution of Medicine/Vaccine/Technology |  |  |  |  |  |  |  |
| 5.3 Reimbursement policy |  |  |  |  |  |  |  |
| 5.4 Use of generic drugs |  |  |  |  |  |  |  |
| 5.5 Other medicines/vaccines/technology issues |  |  |  |  |  |  |  |
| 6.1 Separation of provision and purchasing |  |  |  |  |  |  |  |
| 6.2 Decentralization |  |  |  |  |  |  |  |
| 6.3 Political commitment |  |  |  |  |  |  |  |
| 6.4 Other governance issues |  |  |  |  |  |  |  |
| 7.1 Economic growth |  |  |  |  |  |  |  |
| 7.2 Fiscal capacity |  |  |  |  |  |  |  |
| 7.3 Economic stability |  |  |  |  |  |  |  |
| 7.4 Income inequity |  |  |  |  |  |  |  |
| 7.5 Poverty eradication |  |  |  |  |  |  |  |
| 7.6 Other economic context |  |  |  |  |  |  |  |
| 8.1 Small islands |  |  |  |  |  |  |  |
| 8.2 Remote areas |  |  |  |  |  |  |  |
| 8.3 Other geographic issues |  |  |  |  |  |  |  |
| 9.1 Population size |  |  |  |  |  |  |  |
| 9.2 Aging |  |  |  |  |  |  |  |
| 9.3 Internal and international migration of population |  |  |  |  |  |  |  |
| 9.4 Other population issues |  |  |  |  |  |  |  |
| **10. Climate change** |  |  |  |  |  |  |  |
| **11. political instability** |  |  |  |  |  |  |  |
| **12. other context issues** |  |  |  |  |  |  |  |

Note: This table presents the analytical matrix used to guide country-level evidence mapping in the explanatory evidence review and structured document analysis. It is shown here to illustrate how evidence was organised across thematic domains and countries or areas in the WPR, rather than as a completed coded results table.

**Supplementary Appendix 3.**

**Supplementary figure 3.1 Trends in SDG 3.8.1 across 28 countries in the Western Pacific Region, 2000–2022**


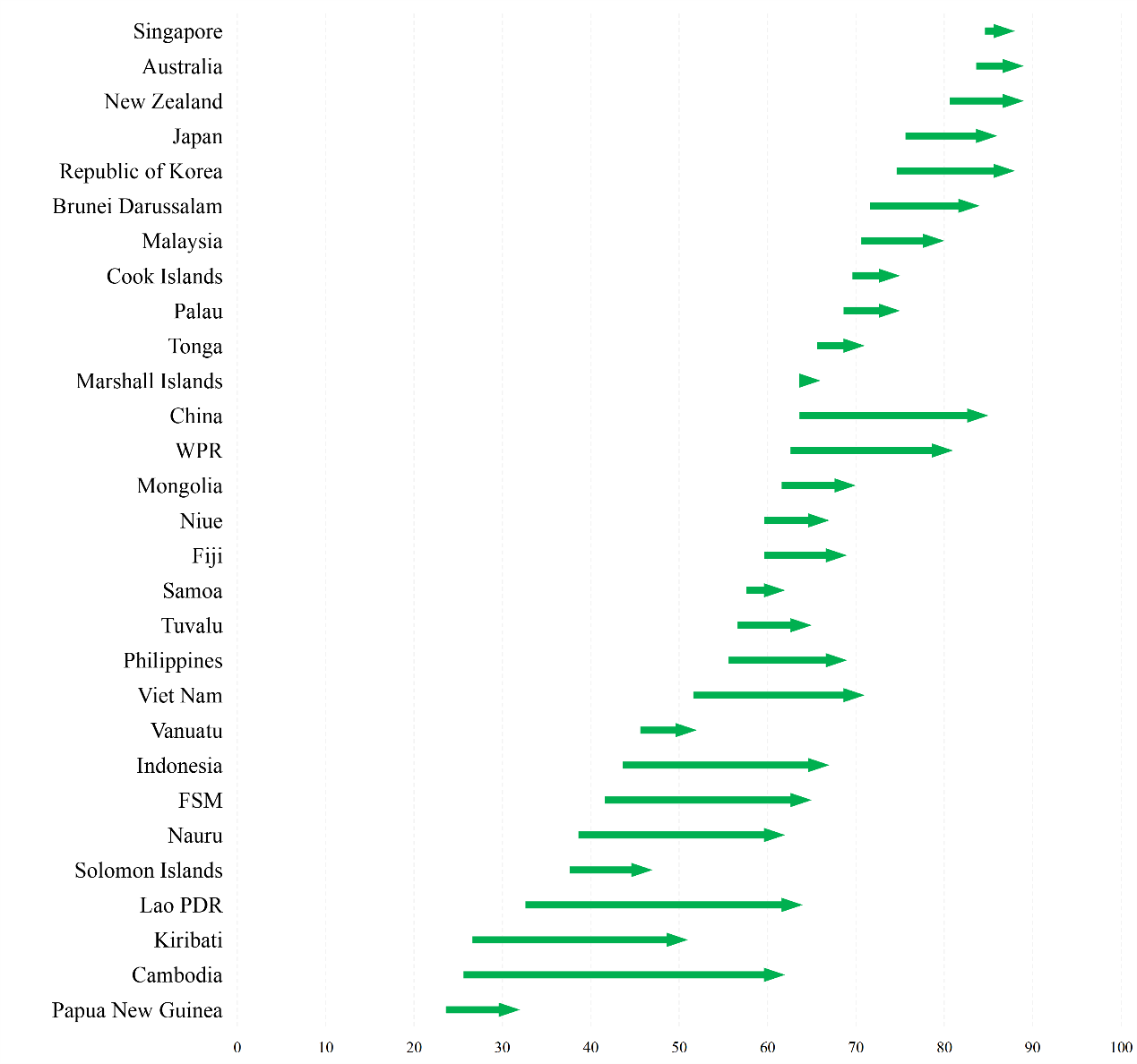


**Supplementary figure 3.2 Trends in RMNCH sub-index across 28 countries in the Western Pacific Region, 2000–2022**


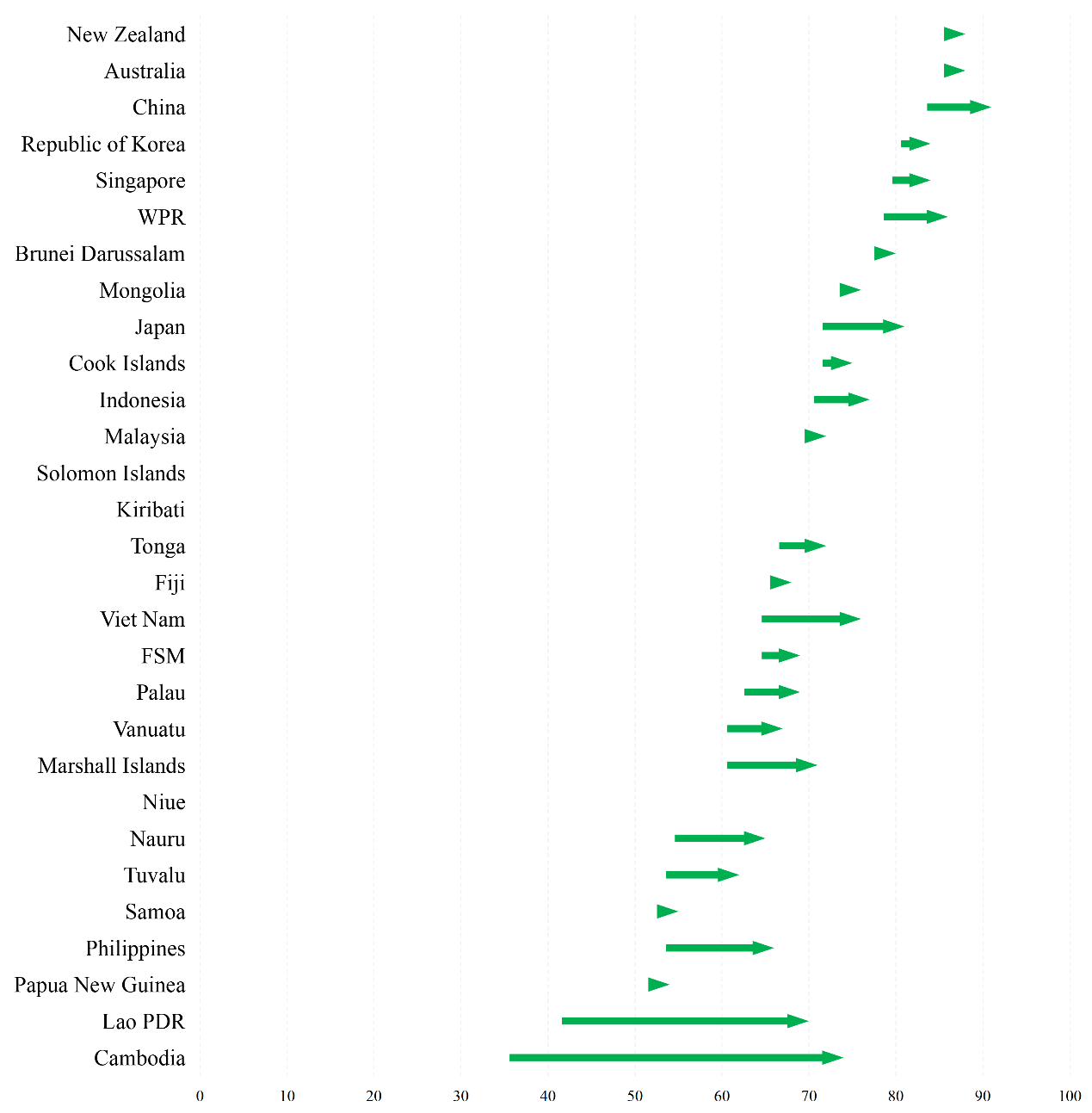


**Supplementary figure 3.3 Trends in infectious diseases sub-index across 28 countries in the Western Pacific Region, 2000–2022**


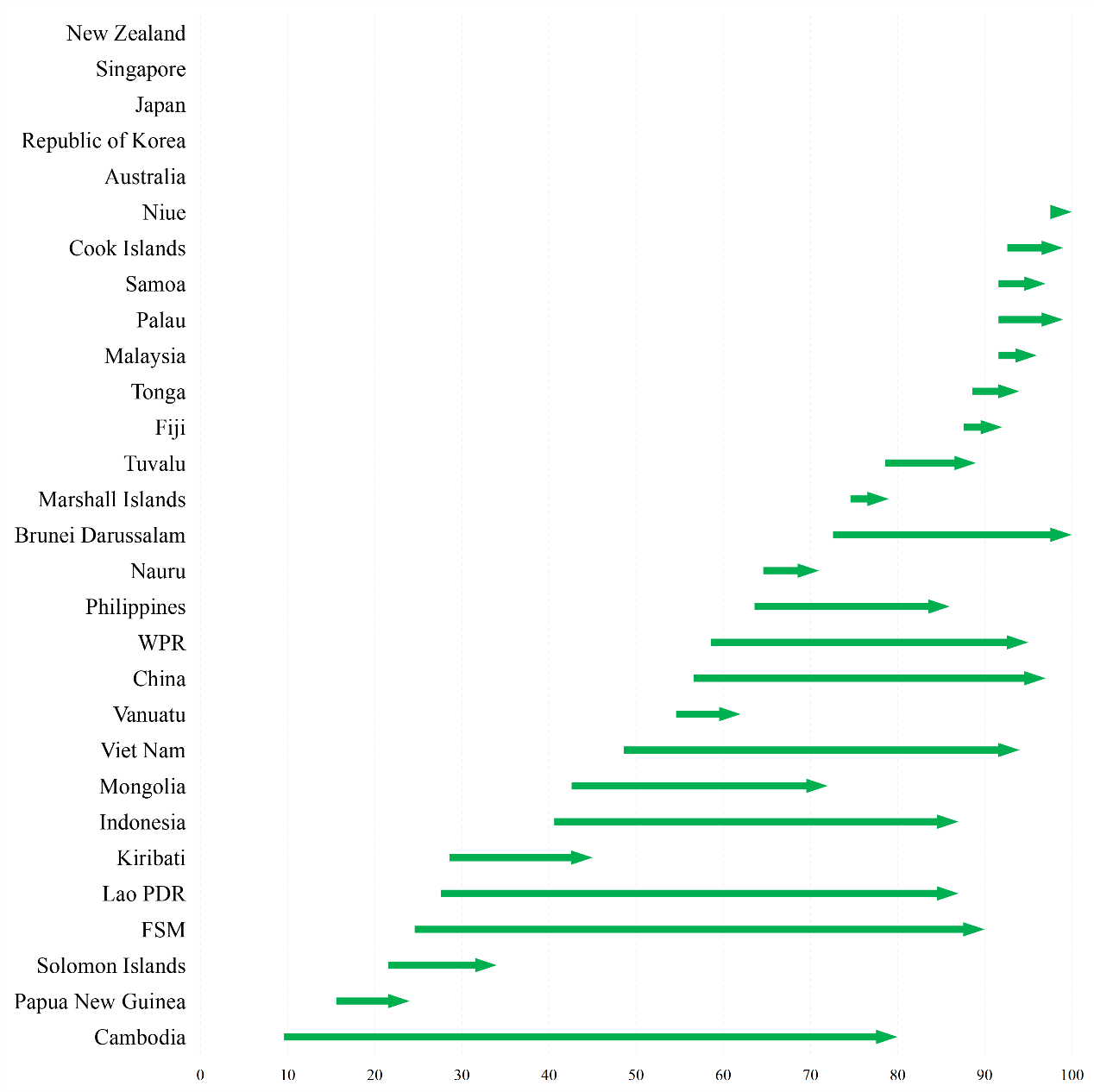


**Supplementary figure 3.4 Trends in noncommunicable diseases sub-index across 28 countries in the Western Pacific Region, 2000–2022**


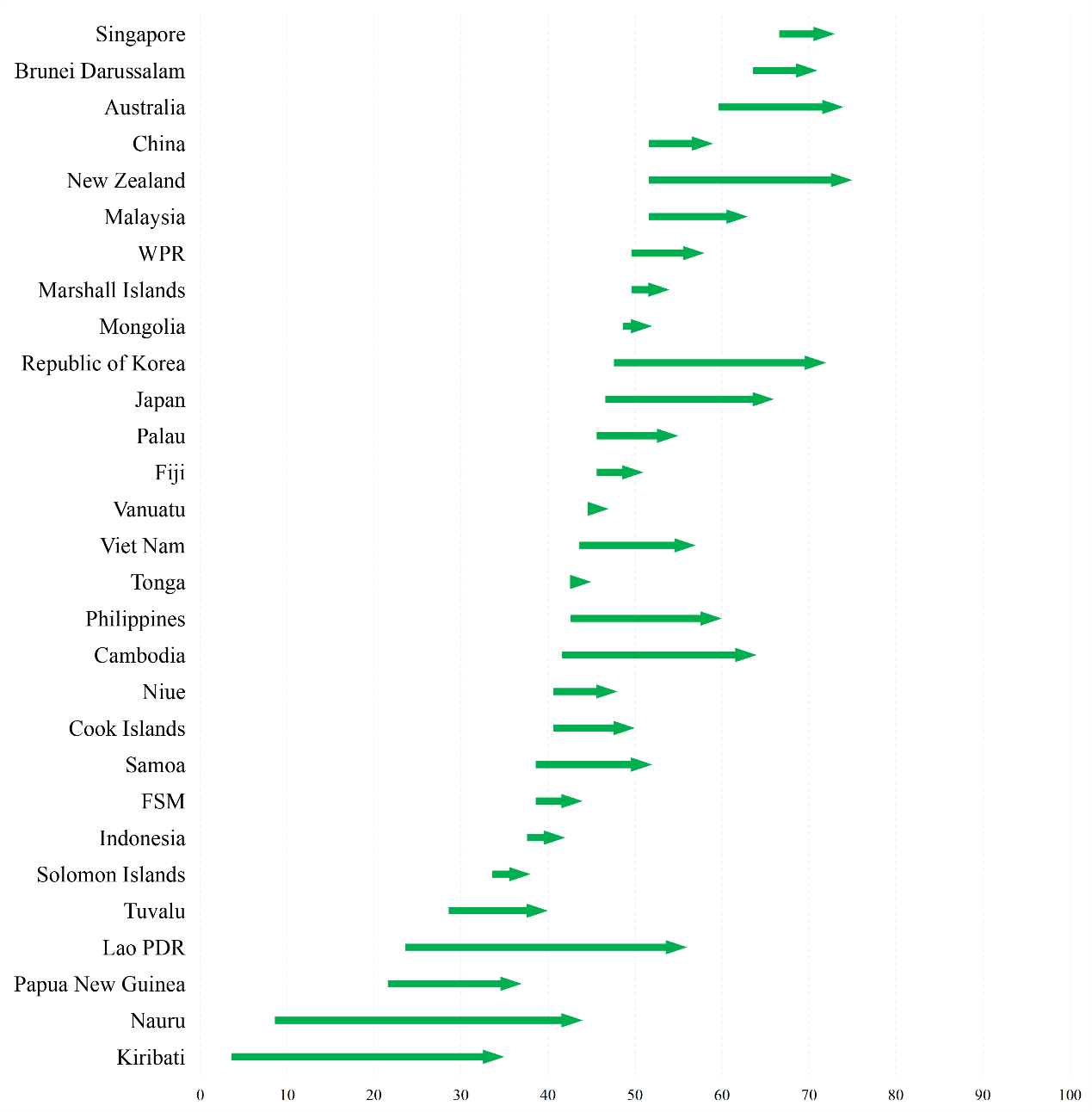


**Supplementary figure 3.5 Trends in service capacity and access sub-index across 28 countries in the Western Pacific Region, 2000–2022**


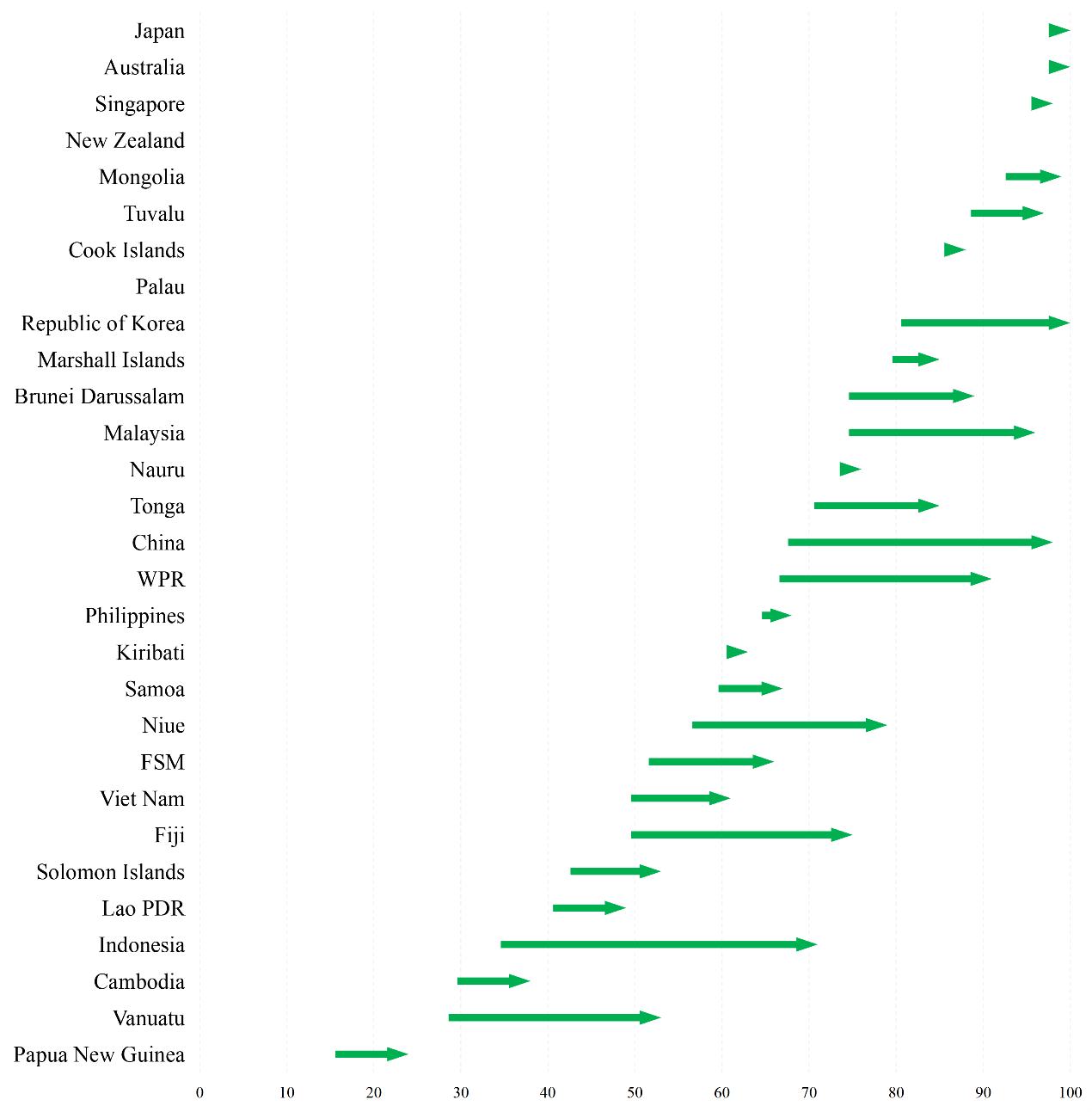

Supplement: supplementary file clean version.docx [file ZGHA_A_2659459_SM9901.docx]
